# Supplementary material for: Cases of trisomy 21 and trisomy 18 among historic and prehistoric individuals discovered from ancient DNA
Source: Nat Commun. 2024 Feb 20;15:1294. doi: 10.1038/s41467-024-45438-1 (PMC10879165; doi:10.1038/s41467-024-45438-1)
Supplement: Supplementary file 1 — Supplementary Information [file 41467_2024_45438_MOESM1_ESM.pdf]

Cases of Trisomy 21 and Trisomy 18 among Historic and Prehistoric Individuals discovered from Ancient DNA

Adam Benjamin Rohrlach[1,2,*], Maïté Rivollat[1,3,4,5], Patxuka de-Miguel-Ibáñez[6,7,8], Ulla Moilanen[9], Anne-Mari Liira[10], João C. Teixeira[11,12,13,14], Xavier Roca-Rada[12], Javier Armendáriz-Martija[15], Kamen Boyadzhiev[16], Yavor Boyadzhiev[16], Bastien Llamas[12,13,17,18], Anthi Tiliakou[1], Angela Mötsch[1,19], Jonathan Tuke[2], Eleni-Anna Prevedorou[20], Panagiota Polychronakou-Sgouritsa[21], Jane Buikstra[22], Päivi Onkamo[9,23], Philipp W. Stockhammer[1,19,24], Henrike O. Heyne[25], Johannes R. Lemke[26,27], Roberto Risch[28], Stephan Schiffels[1], Johannes Krause[1], Wolfgang Haak[1], Kay Prüfer[1]

Supplementary Methods

Section 1 Estimating posterior probabilities of cases of trisomies

Consider detecting a trisomy of chromosome $c \epsilon \{1,..,22\}$. For an individual that does not carry trisomy-c, let the probability that a read maps to chromosome c be p_c, and hence the probability that a read maps to any other chromosome is $1 - p_c$. Let the total number of reads be N, and the number of reads (out of N) that map to chromosome c be X. It follows that a naïve distribution for the number of reads that map to chromosome c would be $X \sim B(N, p_c)$.

However, as X will vary for reasons beyond natural distributional variability, such as sequencing conditions and sample quality among others, we include a prior distribution for p_x such that $p_x \sim Beta(\alpha, \beta)$. The parameters of the prior distribution can then be estimated from the reference data (with outliers removed)[2].

Hence we have that the posterior distribution of the number of reads that map to chromosome c is beta-binomial, denoted $BB(N, \alpha, \beta)$ with probability mass function

$$P(X = x \mid N, \alpha, \beta, c) = \frac{N!}{x!(N-x)!}\left[\frac{B(X+\alpha, N-x+\beta)}{B(\alpha,\beta)}\right],$$

where $B(z_1, z_2)$ is the Beta function of the form

$$B(z_1, z_2) = \int_0^1 t^{z_1-1}(1-t)^{z_2-1}dt.$$

For an individual that carries Trisomy-c, the probability of a read mapping to chromosome c is approximately 50% higher, and is expected to be

$$p_c^T = \frac{3p_c}{2},$$

with probability $1 - p_c^T$ that a read maps to any other chromosome[3].

A beta distribution for the prior distribution of $p_c^T \sim B(\alpha^T, \beta^T)$ can also be found such that the variance of the proportion of reads mapping to chromosome c is unchanged, but such that the expected proportion is increased by 50%, *i.e.*,

$$\frac{\alpha}{\alpha+\beta} = \frac{2\alpha^T}{3(\alpha^T + \beta^T)}$$

and

$$\frac{\alpha\beta}{(\alpha+\beta)^2(\alpha+\beta+1)} = \frac{\alpha^T\beta^T}{(\alpha^T+\beta^T)^2(\alpha^T+\beta^T+1)},$$

which yields that

$$\alpha^T = \frac{3\alpha\left(-3\alpha^2 + 3\alpha(\beta - 1) + 2\beta(3\beta + 1)\right)}{8\beta(\alpha + \beta)}$$

and

$$\beta^T = \frac{3\alpha^3+3\alpha^2(3-9\beta)-8\alpha\beta+4\beta^2(3\beta+1)}{8\beta(\alpha+\beta)},$$

assuming that $\alpha \neq 0$, $\beta(\alpha + \beta) \neq 0$, and

$$\alpha(\alpha^2 + 2\alpha\beta + \alpha + \beta^2 + \beta)(3\alpha^2 + \alpha^2[3 - 9\beta] - 8\alpha\beta + 4\beta^2[3\beta + 1]) \neq 0.$$

Hence, the probability mass function for the trisomy-c case is

$$P(X = x \mid N, \alpha^T, \beta^T, c^T) = \frac{N!}{x!(N-x)!}\left[\frac{B(X+\alpha^T, N-x+\beta^T)}{B(\alpha^T,\beta^T)}\right].$$

Assuming that the probability of a trisomy-c birth, denoted $P(c)$ is unchanged from modern estimates, we have that the normalised posterior probabilities of an individual carrying trisomy-c or not are

$$P(\, c \mid N, \alpha^T, \beta^T, X = x) = \frac{P(c)P(X = x \mid N, \alpha^T, \beta^T, c)}{P(c)\sum_{X=0}^{N} P(X = x \mid N, \alpha^T, \beta^T, c) + [1 - P(c)]\sum_{X=0}^{N} P(X = x \mid N, \alpha^T, \beta^T, c^T)}$$

and

$$P(\, c \mid N, \alpha^T, \beta^T, X = x) = \frac{[1 - P(c)]P(X = x \mid N, \alpha^T, \beta^T, c)}{P(c)\sum_{X=0}^{N} P(X = x \mid N, \alpha^T, \beta^T, c) + [1 - P(c)]\sum_{X=0}^{N} P(X = x \mid N, \alpha^T, \beta^T, c^T)}$$

respectively.

Note that all calculations are performed using log-probabilities, and we use the *LogSumExp* function from the *matrixStats*[1] package to calculate the final posterior probabilities as defined above. All statistical analyses were performed using the R-statistical software package[2].

Section 2 Diagnostic plots for identifying true trisomy cases

In some cases, although strict lab protocols are followed in dedicated ancient DNA (aDNA) labs, sequencing properties can vary between sequencing runs, including relative mapping proportions such as we describe. To distinguish between cases where chromosomal mapping proportions are a true signal of a trisomy, we calculate a set of statistics for each individual, based on the reference data of M individuals.

Consider the proportion on reads that mapped to chromosome c for individual i, denoted $\hat{p}_{i,c}$, out of a total of $N_{i,c}$. For each chromosome, we estimate the parameters of a beta distribution, denoted α_c and β_c, using the full, filtered screening data set. We then calculate the Z-score using the beta-binomial distribution as described in the previous section. That is

$$Z_{i,c} = \frac{\hat{p}_{i,c} - \mu_{i,c}}{\sigma_{i,c}},$$

where μ_c and σ_c are the mean and standard deviation of the beta-binomial distribution for chromosome c, *i.e.*,

$$\mu_{i,c} = \frac{N_{i,c}\alpha_c}{\alpha_c+\beta_c} \text{ and } \sigma_{i,c} = \sqrt{\frac{N_{i,c}\alpha_c\beta_c(\alpha_c+\beta_c+N_{i,c})}{(\alpha_c+\beta_c)^2(\alpha_c+\beta_c+1)}}.$$

We then plot the Z-scores for each individual of interest with a sufficient number of individuals, and preferably from the same sequencing run and site (where possible), looking to identify samples where *only* chromosome c has been enriched.

To estimate the prior distributions of the true mapping probabilities, we filter for samples with more than 10^5 total reads, although we perform trisomy detection on any sample with more than 10^3 total reads. To save computational effort, when calculating the denominator of the posterior probabilities, we use a grid of size 10^5, starting at five standard deviations below the mean, and ending at five standard deviations above the mean.

Section 3 Data Processing

Our data consists of a total of 17,269 sequencing libraries from 13,153 individuals that were shotgun sequenced on the Illumina platform almost exclusively in single-read mode with 76 base pair length. Approximately half of the sequencing libraries were prepared using a variant of a double-stranded library protocol[3] and another half with a variant of a single-stranded library protocol[4]. An enzyme treatment to remove ancient DNA associated cytosine deamination before sequencing was applied to the majority of double-stranded libraries and around 20 percent of the single-stranded libraries[5]. All libraries were identified by unique index-pairs[3] and reads were assigned to a library when indices matched the expected indices with up to one mismatch per index. Illumina adapters were trimmed using leeHom version 1.1.5 with parameter `−ancientdna`[6]. Sequences were aligned with BWA version 0.7.12 with parameters `-n 0.01 -o 2 -l 16500`[7]. Further processing, including the counting of sequences that aligned to autosomes, was carried out with samtools version 1.3. Ancient DNA libraries can be low-complexity leading to independent observation of identical fragments due to PCR duplicates. To mitigate the effect of PCR duplicates we only consider the first alignments to any 5’ coordinate and discard all following alignments matching this 5’ coordinate. After removing duplicates, sequences with a minimum sequence length of 35 and a mapping quality of 25 were counted for all autosomes.

Section 4 Site and Sample Descriptions

Note: All genetic sexes were assigned using sequence data.

Yunatsite, Pazardzhik, Bulgaria (YUN)

Tell Yunatsite, also known as Ploskata Mogila (the Flat Mound), is among the largest tell settlements in Bulgaria[8,9]. Its largest diameters are 110 x 100 m and it rises 12 m above the modern surface. The site is situated in southern Bulgaria, at the western periphery of the Upper Thracian Lowland, 6 km to the northwest of the town of Pazardzhik and ca. 1 km to the southwest of the village of Yunatsite (Fig. 4). In the past, this was an extremely favourable location. It was established on a low terrace on the right bank of the Topolnitsa River, near its confluence with the Maritsa River. It was located in a fertile plain area, surrounded on three sides by mountains: Rhodope Mountains to the south (ca. 16 km away), Rila and Ihtimanska Sredna Gora Mountains to the west (ca. 25–30 km away) and Sashtinska Sredna Gora Mountain to the north (its southern slopes are ca. 7–8 km away) (Figs. 3, 4).

The site was located at an excellently situated strategic point and communicative centre. The Upper Thracian Lowland is connected by suitable routes to the west to the Struma Valley and Sofia Field and from there, through the valleys of the Nishava and Iskar Rivers – to the Danube. It is not a surprise that Via Diagonalis, one of the main roads of the Roman Empire, passed about 500 m to the south of the tell. The Maritsa River provided an easy connection to the central parts of the Upper Thracian Lowland to the east and thence to the Aegean coast to the south. The Mesta Valley divides the Rila and Rhodope Mountains and can also be used as an alternative route to the north Aegean coast.

The favourable location, providing occupation for various ethnic groups in a period of 6000 years, resulted in the accumulation of the deposits and the formation of the tell. In Yunatsite, 24 urn infant burials were found, under house floors. Urn infant burials have been found in four other EBA sites in Bulgaria[10.]

The individual YUN039/Burial 3 was dated to the Early Bronze Age. The age was estimated to be 6 months old and the remains were found in an urn-burial in the basement of the dwelling, Level Б XI, building 12A.

Unfortunately, no images exist of the skeleton, and the complete skeleton is no longer accessible, after being lost during transfer to Russia. Some elements of the skeleton can be found at the Regional Museum of History in Pazardzhik. However, from the report from the original anthropologist, the bones were apparently well-preserved.

Dating of the skeleton:

- YUN039: 2898-2700 BCE (MAMS-45495).

Skeletal elements recovered:

- YUN039: Fragmentary cranial remains, paired humerii, radii, fragments of paired ulnar, femoral, paired tibia, fragments of the fibula, pelvis, scapula.

Lazarides, Aegina, Greece (LAZ)

The Mycenean settlement at Lazarides, on the eastern side of the island Aegina, thrived during the palatial period, the 14th and the 13th c. BCE[11]. The available evidence, however, points to an earlier installation at the site, dating to the MH III/LH I period, whilst the use of the nearby cemetery covers the LH IIIA2early - LHIIIB2/IIIC period. Presently only built chamber tombs have been discovered, which, though looted, provide abundant information concerning the status of the deceased and the dynamic nature of the site. In addition to the organised cemetery, intramural burials of mostly children were found in shallow pits, although a few other types were found. Among these was found a tiny, carefully made cist grave that was excavated in 2010, and dated to the 13th c. BCE, on the basis of its location beside a retaining wall that was rebuilt during this time. The skeletal remains belonged to an infant that was wearing a necklace of 93 beads of several shapes and size, of glass paste and fayence, along with four small cylinders of carnelian. The find was transported with the soil of the tomb to the laboratory of the Museum of the Department of Archaeology and History of Art, University of Athens, where it was carefully cleaned and examined by Dr M. Rockenbucke and Dr E. Prevedorou, respectively[11,12].

The studied individual was buried in the settlement along with a beaded necklace[11,12]. Based on current preliminary evidence, at Mycenaean Lazarides, the very young juveniles, i.e., perinates, neonates, and young infants, were buried in shallow pits inside the settlement. However, given the small dataset and the limited skeletal evidence from the nearby cemetery, this observation should remain tentative.

Infant LAZ-019 was approximately 1 year old (12-16 months) at the time of death. Cranial and postcranial lesions suggested severe anemia, possibly of genetic origin, and infectious comorbidities. For excavation, bone preservation and analysis, see the detailed bioarcheological study published by Prevedorou, 2010-2011[12].

The remains are currently stored at The Ephorate of Antiquities of Piraeus and Islands, Hellenic Ministry of Culture and Sports. The Director of the excavation and the individual in charge of the material is Professor Naya Polychronakou-Sgouritsa, University of Athens.

Dating of skeleton:

- LAZ019: 1398-1221 BCE (MAMS-47525)

Skeletal elements recovered:

- LAZ019: The skeleton was relatively complete, but missing the upper limbs, part of the pelvis, and most of the hand and foot bones.

Alto de la Cruz, Navarra, Spain (CRU)

Alto de la Cruz as well as Las Eretas belong to the first proto-urban centres in northern Iberia, emerging at the transition between the Bronze Age and the Iron Age (c. 900-800 BCE). Their fortification walls, constructed with mud bricks and/or stone, protected a densely built settlement space, formed by rows of attached rectangular buildings, separated by passages and narrow streets, which in some cases were stone paved. The size of the community was probably limited to a few hundred individuals, but part of the population could also have lived in open settlements close to these centres. Given the similarity of the house plans and the domestic remains, social differences seem to have been limited, especially during the early phases.

Alto de la Cruz, placed in the municipality of Cortes de Navarra, is a c. 0.75 ha large tell settlement with an up to 6 m stratigraphic record which formed from the final Bronze Age until the end of the Early Iron Age (c. 950-400 cal BCE). The settlement has been excavated extensively in several campaigns since 1947[13-16]. Infant burials of a very young age were recognised since the beginning of the excavations under the floor levels of rectangular houses. The published information suggests that a few dozen of these infants were identified, but unfortunately, they were not documented in a systematic way until the 1980s, implying that we only know the stratigraphic and archaeological context of a few of them. All 29 infant remains sampled at the Archaeological Deposit of the Government of Navarra provided more or less well-preserved genetic material. The dominant burial custom in this region at the time for adults was cremation, however, some perinates and infants received intramural burials.

CRU001/Cruz 1 and CRU013/Cruz 13 probably belonged to the latest occupation phase (settlement I), whereas CRU024/Cruz 24 was found in a building of the first Iron Age settlement (IIIb). The perinatal individual CRU024/Cruz 24 is exceptional because it died at a premature age (approximately 27 weeks) and was found in a special archaeological context, which was well documented and carefully excavated during the excavations carried out in 1986-87. This individual was buried with comparatively rich grave goods, formed by three bronze rings and one *Glycymeris glycymeris* shell (probably brought from the Mediterranean Sea)[16]. Moreover, the individual seems to have been buried with three complete sheep/goats in the central part of a large, rectangular building. It is the only house excavated at Alto de la Cruz in which a large, decorated fireplace has been found[16]. No other individual was buried in this building, which seems to have been a place of ritual[17]. Overall, bone preservation was quite good, although the number of elements which could be recovered for each skeleton varied from nearly complete (CRU024) to relatively few (CRU013).

The remains of all three individuals are under the care of Dr Jesús Sesma, Archeology Section of the Historical Heritage Service, in the Archeology Warehouse, Administrative Archive Building (Pamplona-Zaragoza Highway, km. 1, Cordovilla) (Navarra, Spain), under the ownership of the Foral Government of Navarra, Spain (jesus.sesma.sesma@navarra.es).

Dating of skeleton:

- CRU001: 800-400 BCE (dated via archaeological context)
- CRU013: 800-400 BCE (dated via archaeological context)
- CRU024: 779-549 BCE (MAMS-55002)

Skeletal elements recovered:

- CRU001: Fragmentary cranial remains; the occipital bone is almost complete. Maxilla and mandible. Humeri, ulnae and radii. Both scapulae, a right clavicular fragment, several ribs and some vertebrae. Left ilium and pubis. Fragments of both femurs, left tibia, and a fragment of the right tibia.
- CRU013: Fragmented cranial remains. Left hemiarch axis. Left scapula. Both humeri. Left tibia. Fragment of long bone with morphological alterations, probably a fibula.
- CRU024: Almost complete skeleton, missing the phalanges of the hands and feet.

Las Eretas, Navarra, Spain (ERE)

Las Eretas, in the municipality of Berbinzana, is another Early Iron Age tell site, located c. 80 km north-northwest of Alto de la Cruz. Approximately 15% of this 0.5 ha large settlement was excavated between 1991-1996 and in 2000, revealing a series of rectangular buildings protected by a fortification wall. In this case, mud brick walls were erected on stone plinths. Remains of six children were found in five burials located in two of the four totally and four partially excavated buildings. They were carefully excavated and the sediment was sieved. As with Alto de la Cruz, the dominant burial custom in this region at the time for adults was cremation, however, some perinates and infants received intramural burials.

Infant burial ERE004 was found in 1995 under a large stone block which had been intentionally placed in the South-Western corner of a large rectangular building. The skeleton was recognised as very immature and gracile in the initial anthropological study[18]. This might be caused by the young age of the child or a delayed intrauterine development (IUGR). Bone preservation at the site was quite good, although many bones were fragmented.

The remains of ERE004 are under the care of Dr Jesús Sesma, Archeology Section of the Historical Heritage Service, in the Archeology Warehouse, Administrative Archive Building (Pamplona-Zaragoza Highway, km. 1, Cordovilla) (Navarra, Spain), under the ownership of the Foral Government of Navarra, Spain (jesus.sesma.sesma@navarra.es).

Dating of skeleton:

- ERE004: 791-776 BCE (MAMS-55004)

Skeletal elements recovered:

- ERE004: Mostly complete. However, the left upper and the tibia of the right lower limb bones were not preserved, as well as most phalanges.

Helsinki Senate Square, Finland (HKI)

Between the 1640s and 1790s, the Helsinki city church and cemetery were located on the site of today's Senate Square. The buildings were demolished in the early 19th century, and the area

was left under the present market square and streets[19]. Part of the cemetery has been examined in archaeological excavations that have revealed hundreds of graves. The graves were excavated in the field by experienced archaeologists. Grave 13/HKI002 belonged to a full-term child. The grave was located in the eastern part of the cemetery, close to the cemetery fence, in an area reserved for children. The area contained five layers of graves, older burials deeper in the ground. Grave 13/HKI002 was located in the second layer from the top, and a coin from the year 1667 was found in the grave fill. These details, as well as the dress accessories, suggest that the grave likely dates to the 18th century – at least it cannot be older than the coin or the burials beneath it.

The infant was buried in a wooden coffin. The grave also included bronze pins and decorative bronze flowers. During the 17th century, it was common to dress the deceased in formal clothing. At the turn of the 17th and 18th centuries, there was a shift from the use of actual costumes to funeral attire and shrouds, which were often fastened by pins. Children's funeral costumes followed the same trend as adult costumes, but their heads could have been decorated with floral crowns[20], as in the case of grave 13/HKI002.

The infant was buried in the churchyard according to the standard practices of the period. The burial also shows that the deceased was carefully prepared for the grave by dressing and decorating them. Although disability may have been a reason for social exclusion in the Post-Medieval Period, recent historical research has emphasised that disabled individuals were not considered a problem but seen as active and important members of families and society during the period[21]. The preservation of bones is generally poor in Finland because of the acidic soil. The skeleton of HKI002 was not complete, likely because of the acidic soil, but the remaining bones were quite well preserved. These remains have been reburied.

Dating of skeleton:

- HKI002: 18th century CE (dated via archaeological context)

Skeletal elements recovered:

- HKI002: Fragmentary cranial remains, vertebrae and costae.

Supplementary Tables and Figures

Supplementary tables

*Supplementary Table 1: Reported osteological observations and potential vitamin deficiencies for individuals carrying trisomy 21, based on direct bone observations (individuals CRU013, HEL002, LAZ019) or on respective reports (CRU001, CRU021, ERE004, YUN039). *indicates markers of potential interest but with no direct association to trisomy 21.*

Osteological Marker	CRU001	CRU024	ERE004	HKI002	LAZ019	YUN039
Orbital roof porosity[22]	No	N/A	No	N/A	Yes	Yes
Mandible porosity[22]	Yes	N/A	N/A	Yes	Yes	Yes
Upper palate porosity[22]	No	N/A	N/A	N/A	Yes	Yes
Frontal/parietal bone porosity[22]	No	N/A	No	Yes	Yes	Yes
Porosity or striae in the occipital squama[22]	No	Yes	No	Yes	Yes	Yes
Irregular bone growth on the *pars lateralis occipitalis*[23]	Yes	Yes	No	Yes	?	N/A
Occipital protrusion[24]	Yes	No	N/A	N/A	N/A	No
Inner ear bone malformation[25]	N/A	N/A	No	Yes	N/A	N/A
Striae and pitting of the lower long bones*	No	N/A	No	N/A	N/A	Yes
Striae and pitting of the Ilium*	No	N/A	No	N/A	N/A	Yes
Femur bending/flaring[26]	No	N/A	No	N/A	Yes	Yes
Indications of vitamin C deficiency[26]	No	N/A	No	Yes	No	Yes
Indications of vitamin D deficiency[26]	No	N/A	No	N/A	No	Yes
Enamel Hypoplasia[27]	N/A	N/A	N/A	Yes	N/A	N/A

Supplementary Table 2: Age-at-death estimates in weeks for CRU013 (Edwards syndrome) using different methods of estimation on different skeletal elements.

Measurement	mm	Fazekas & Kósa (1978)[28]	Jeanty (1983)[29]	Scheuer *et al.* (1980)[30]
		Age	Age	Age
Maximal length of right humerus	64.5	40	40	38.22
Distal width of right humerus	11.4	30	-	-
Maximal length of left humerus	65	40	40	38.45
Maximal length of right femur	>71.1	>38	>38	>37
Maximum length of left tibia	58.6	37	34	36.12

Supplementary figures

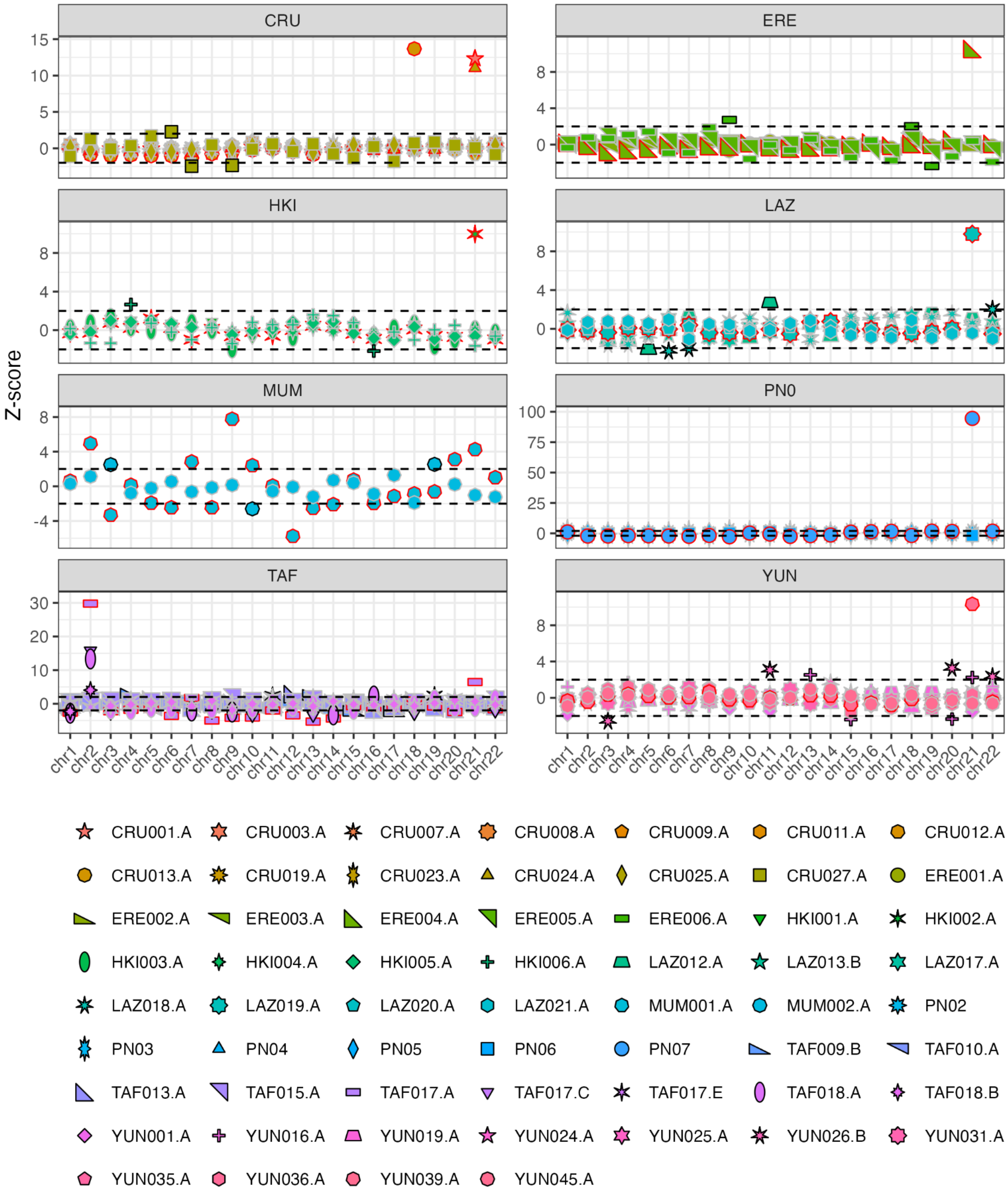

Supplementary Figure 1: Diagnostic plot showing the beta-binomial Z-scores of the chromosome mapping probabilities for all positive cases on the y-axis, and the chromosomes on the x-axis. Red outlines indicate individuals that were initially flagged as positive, and black outlines indicate a significant z-score for a chromosome. Note that while the individuals MUM001 and TAF017 were initially flagged as positive, we were able to observe that it was simply a library that performed abnormally for many chromosomes. The plot is faceted by sample sites, with each facet containing up to 10 additional individuals from each possible site.

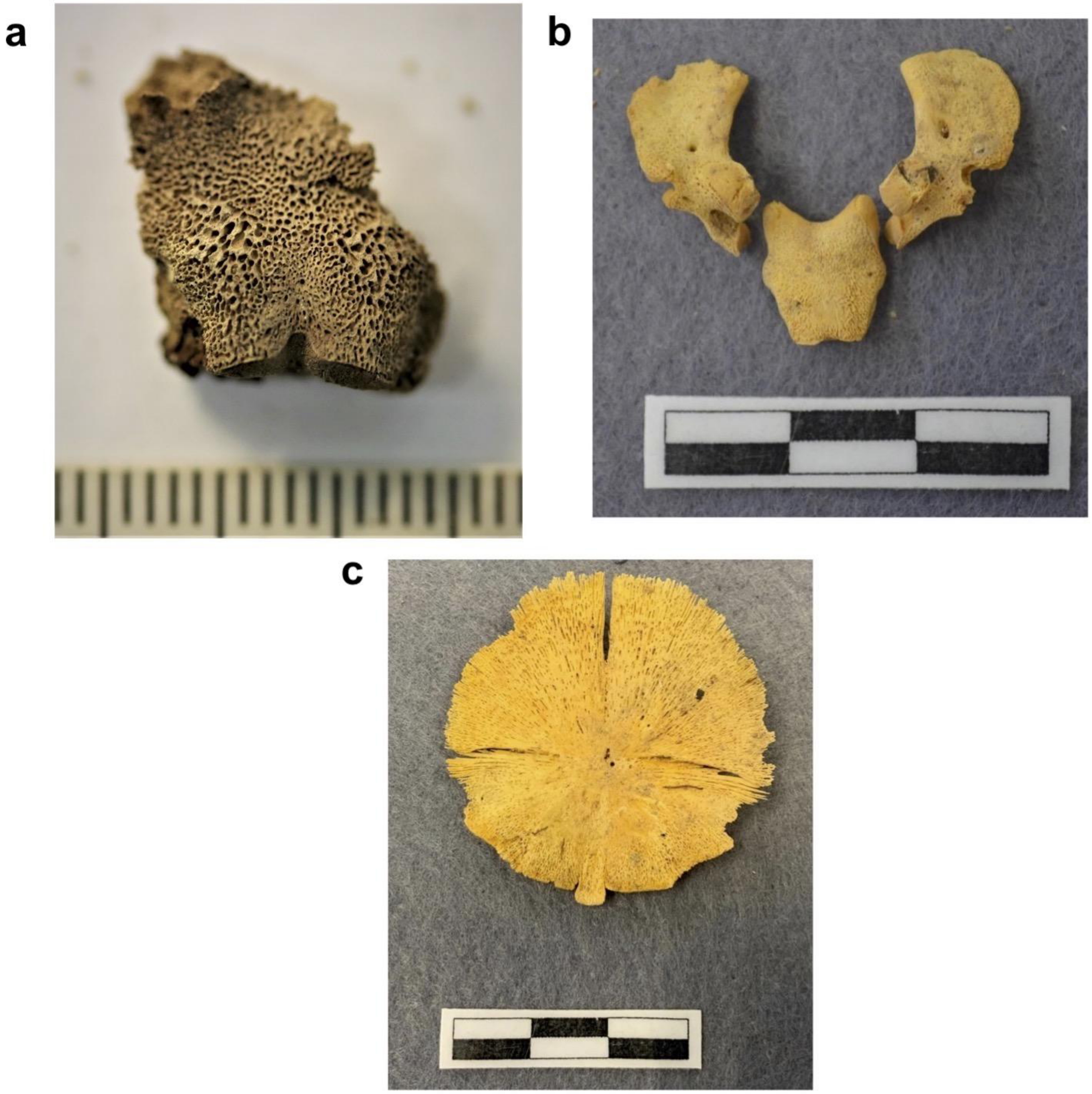

Supplementary Figure 2: (a) Porosity in a fragment of the bars basilaris (inferior) of HKI002 (Helsinki), (b) Irregular bone growth on the unfused pars basilaris and both pars lateralis (inferior), slight exostosis at the jugular end, with evidence of a fissure in the left condylar area, and (c) internal view of the protrusion on the pars squama of the occipital bone from CRU024 (Alto de la Cruz). Photographs by (a) Anne-Mari Liira, (b,c) the Government of Navarre and J.L. Larrion.

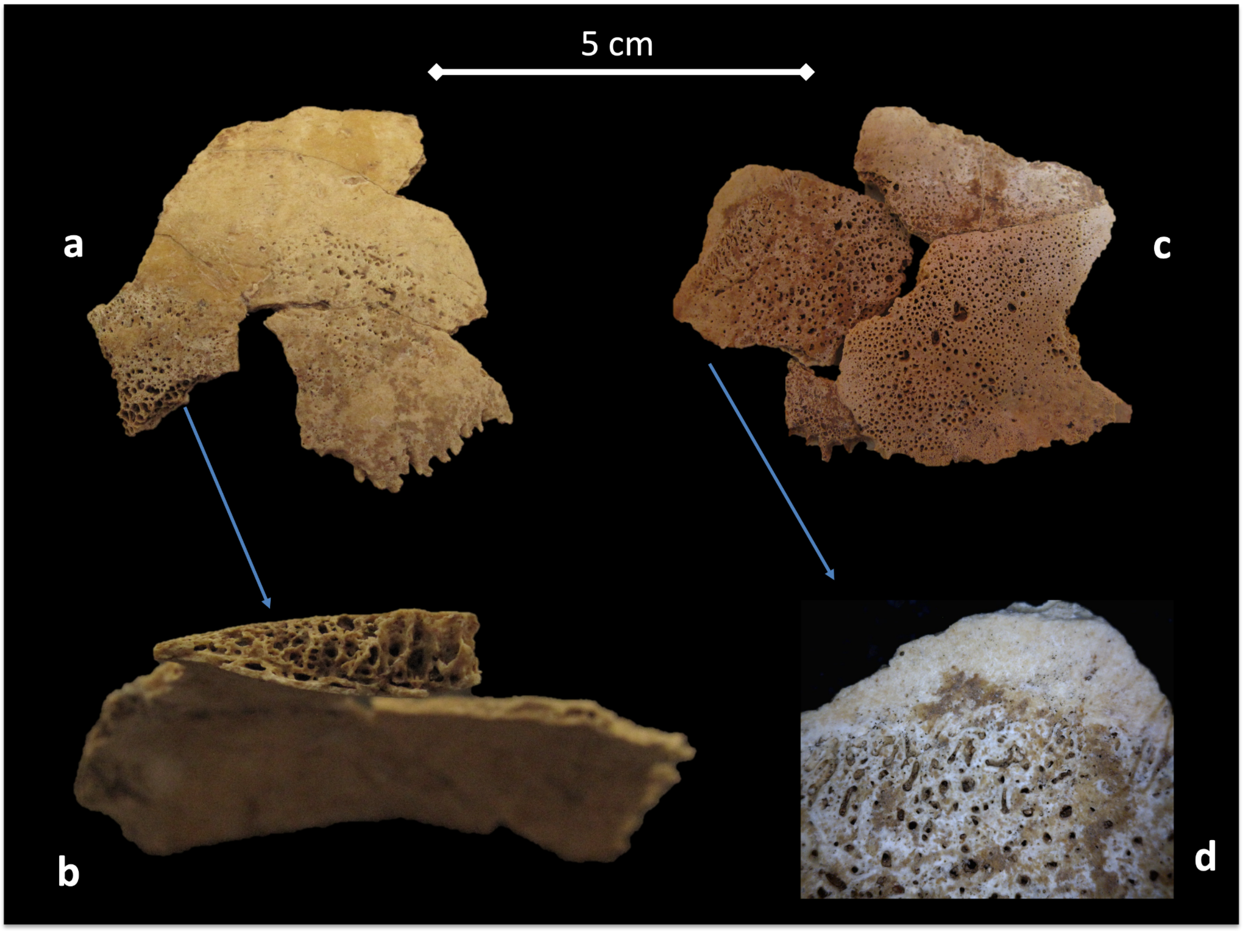

Supplementary Figure 3: Porotic hyperostosis with diploic expansion observed in infant LAZ019: a) ectocranial surface of left parietal bone, b) parietal section, c) ectocranial surface of right parietal, and d) magnified view. Photographs by E. Prevedorou.

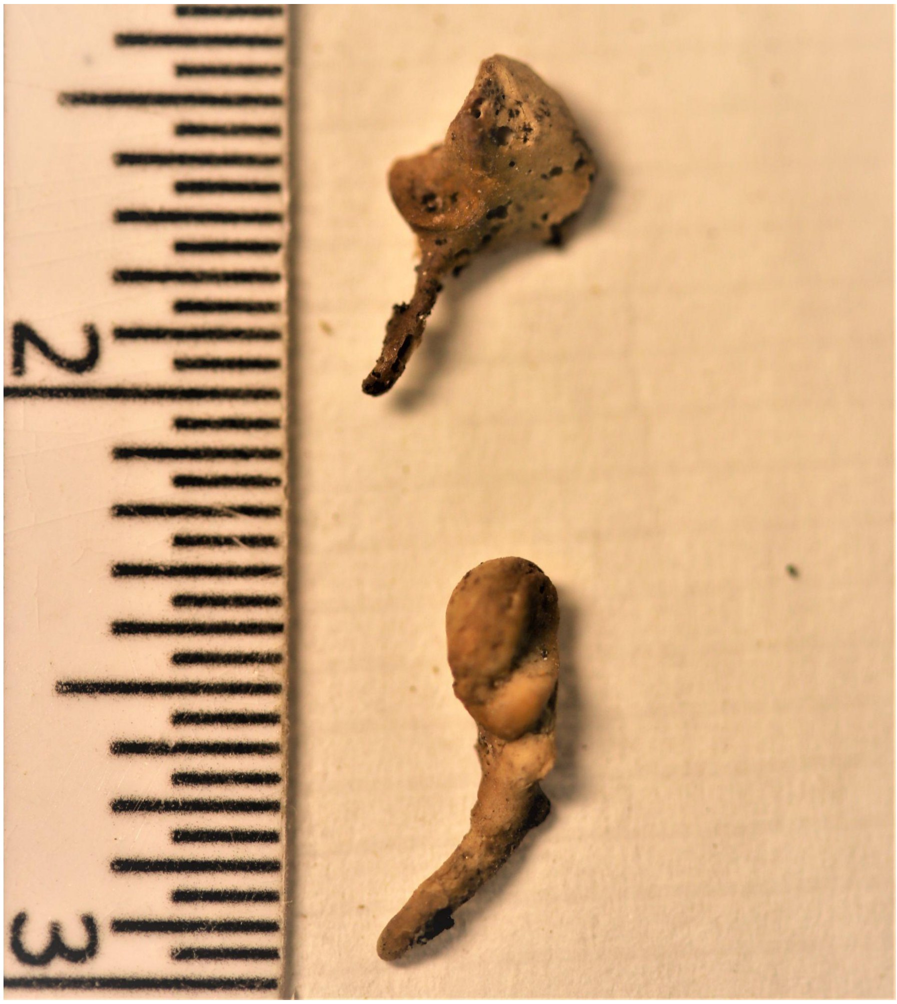

Supplementary Figure 4: Preserved ossicle bones (the left incus (top, lateral) and left malleus (bottom, anterior) of HKI002 (Helsinki), indicating malformation of the incus. Photograph by Anne-Mari Liira.

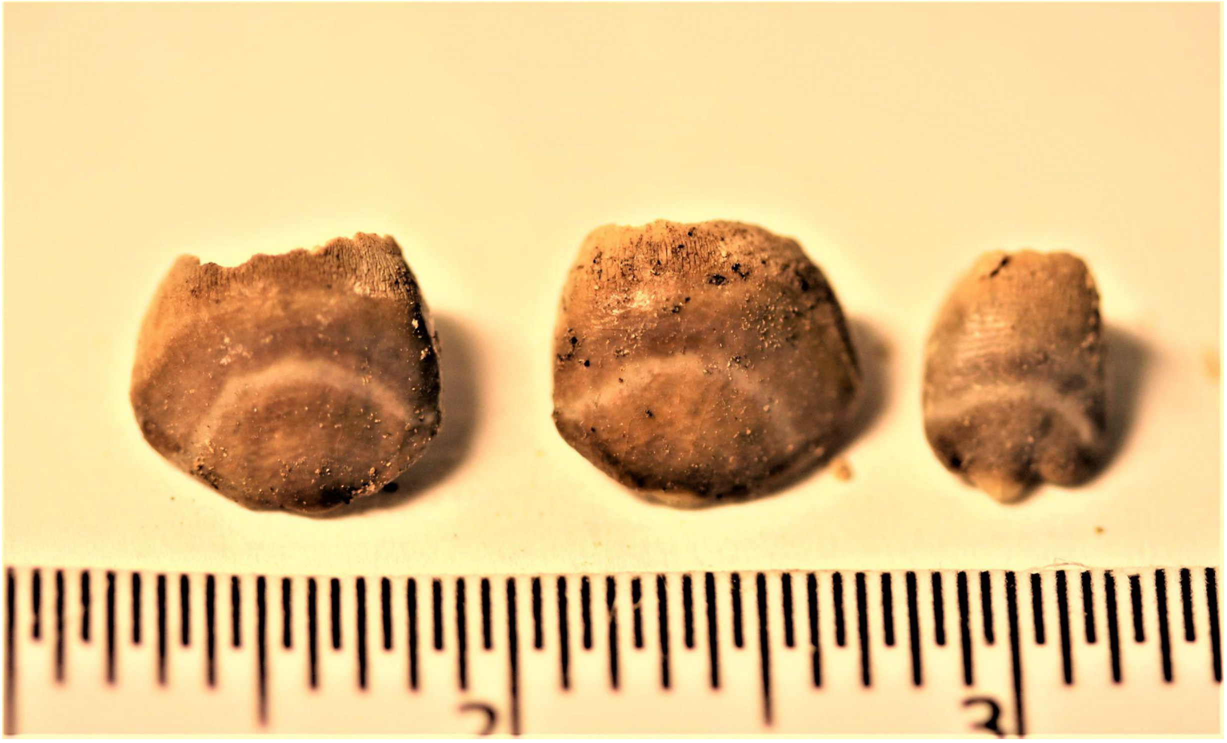

Supplementary Figure 5: White curved lines on the superior deciduous incisors (teeth 51, 61 and 62, labial view) of HKI002 (Helsinki), indicating dental defect, possibly dental enamel hypoplasia. Photograph by Anne-Mari Liira.

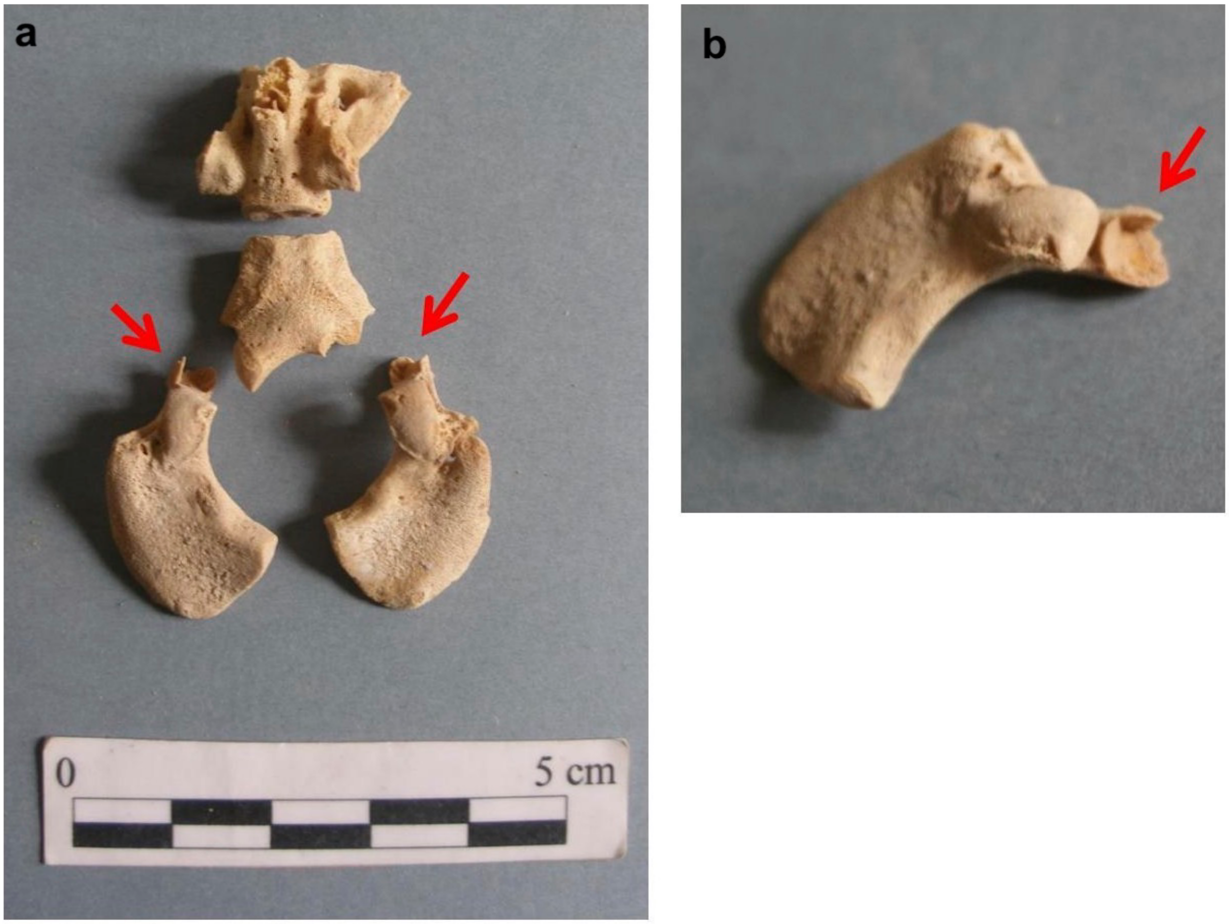

Supplementary Figure 6: Flange at the jugular end of the pars lateralis of CRU001 (Alto de las Cruz). (a) inferior view of both pars lateralis and pars basilaris of the occipital bone and of the sphenoid bone, (b) inferior view of the right pars lateralis. The red arrows indicate (a,b) additional bone growth on the jugular limb of the pars lateralis*.*

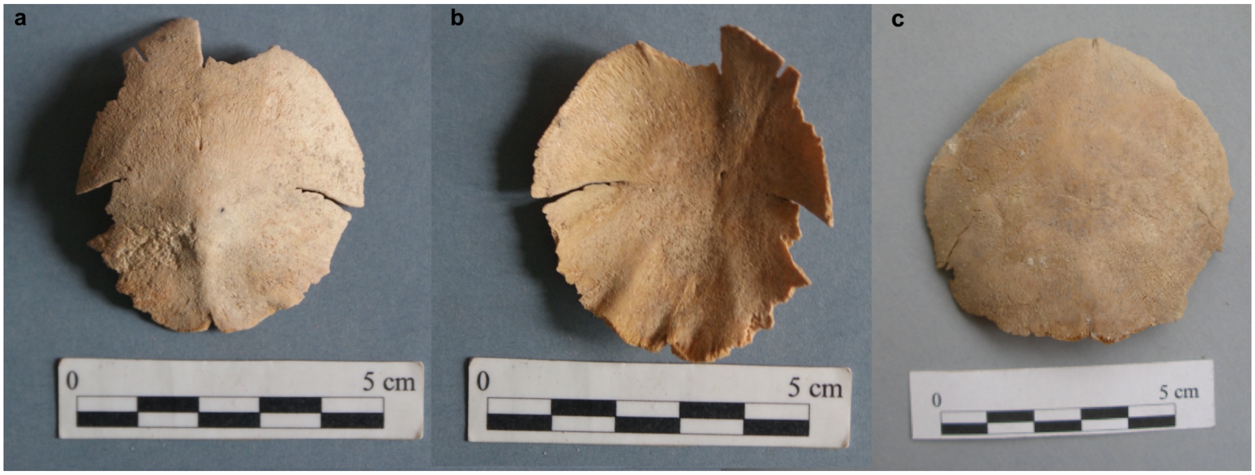

Supplementary Figure 7: Protrusion of the occipital squama of CRU001 (Alto de la Cruz), (a) external view, and (b) internal view compared to (c) the external view of the occipital squama of a similarly-aged individual recovered from Alto de la Cruz (who presented no evidence for trisomy 18 or 21). Photographs from the Government of Navarre and J.L. Larrion.

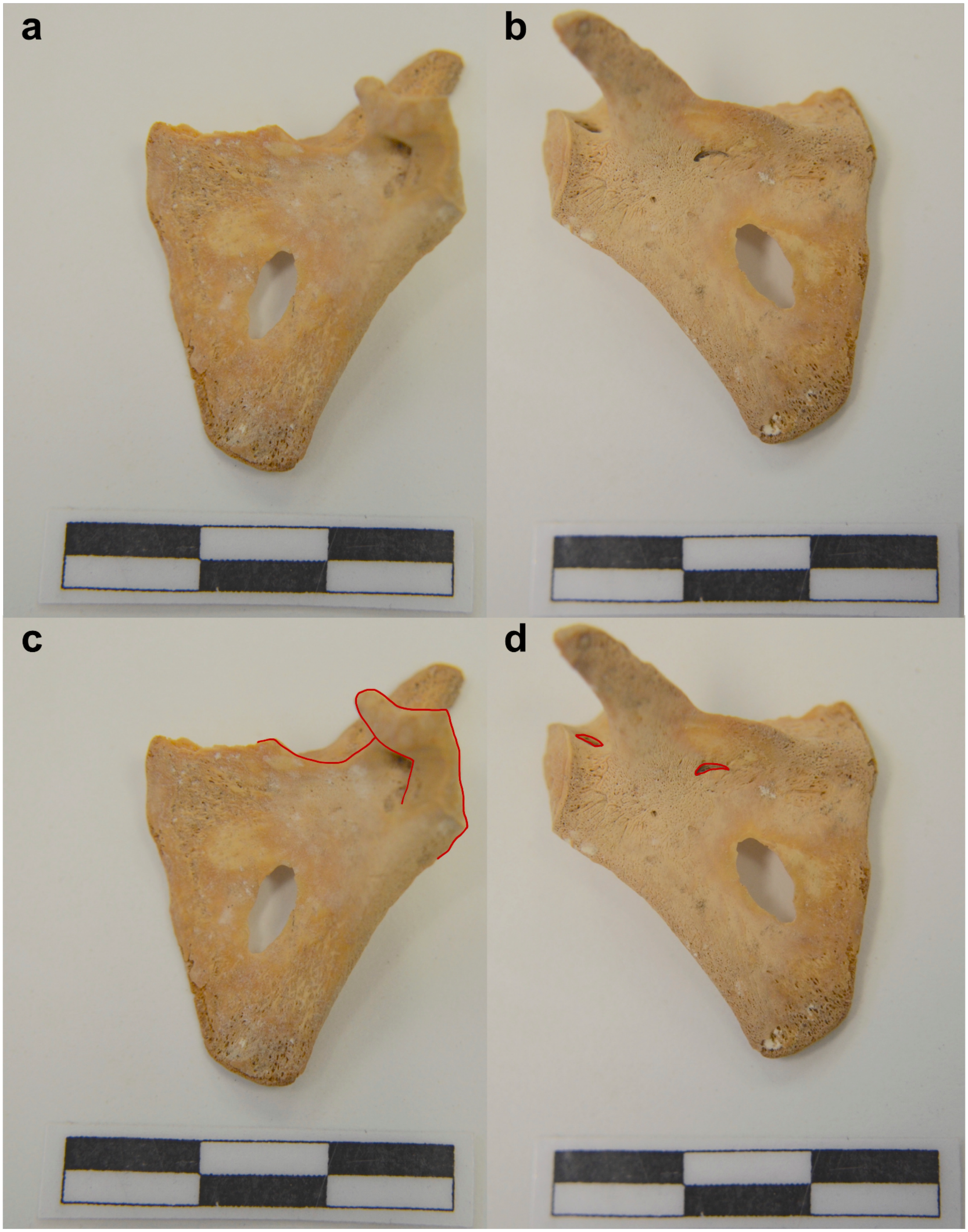

Supplementary Figure 8: anterior (a,c) and posterior (c,d) views of the left scapula of CRU013 (Alto de la Cruz), with ossification defect in the body and alterations indicated in red (b,d). Photographs from the Government of Navarre and J.L. Larrion.

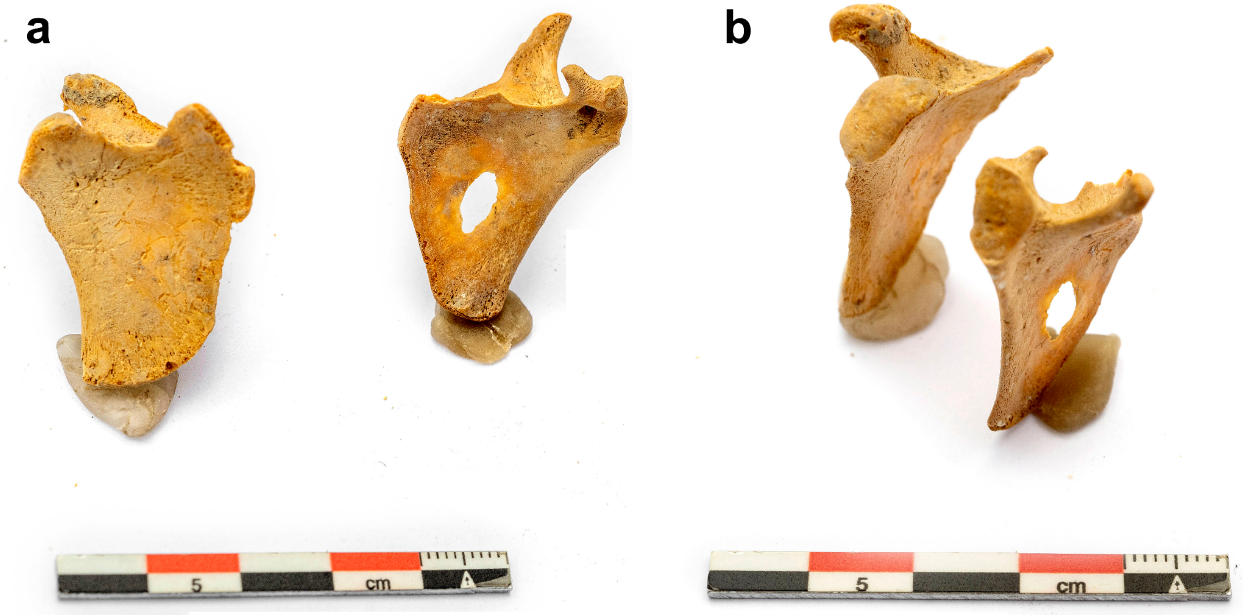

Supplementary Figure 9: A comparison of the (a) ventral view and the (b) lateral view of the left scapula of CRU013 (right in both) and a normal right scapula from an individual of a similar age (left in both), also found at Alto de la Cruz. Photographs by Patxuka de Miguel.

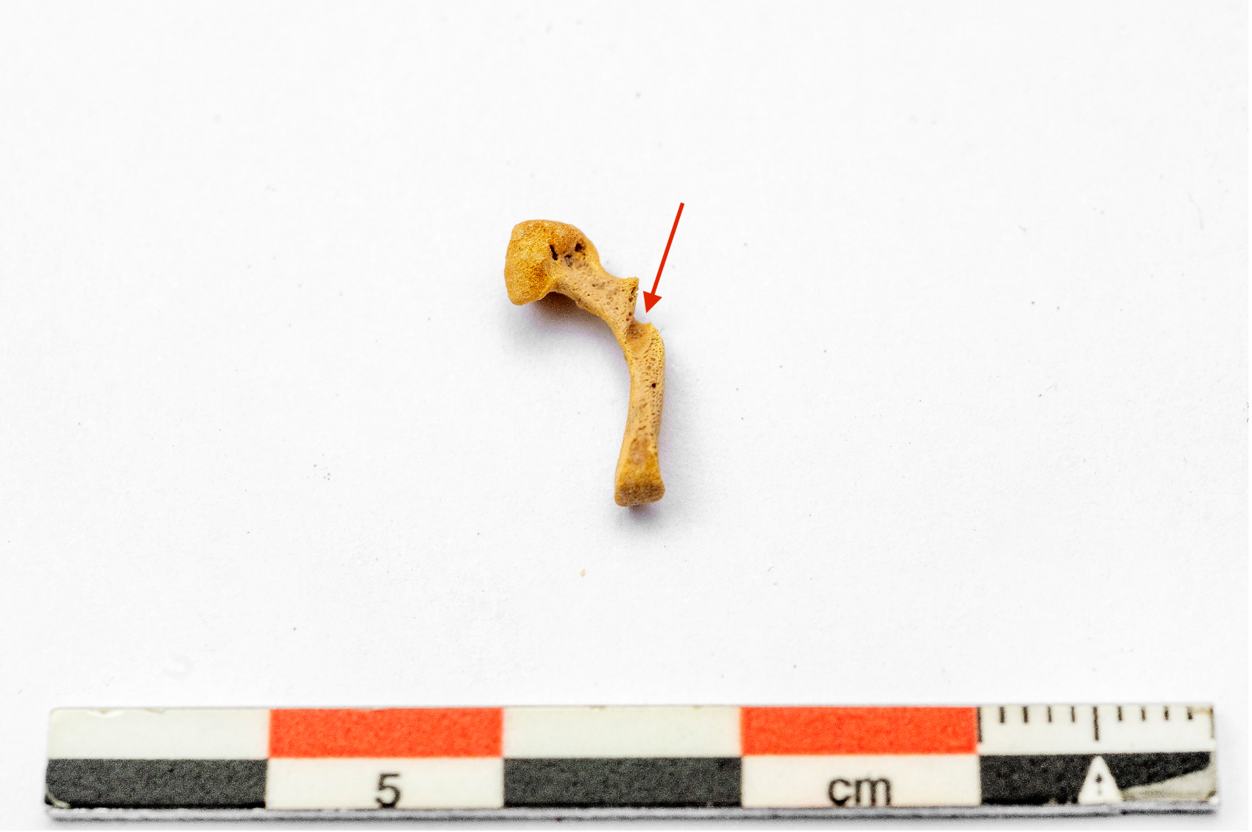

Supplementary Figure 10: porosity of the hemivertebra axis, left side, inferior view, of CRU013 (Alto de la Cruz) with the morphological alteration indicated by the red arrow. Photograph by Patxuka de Miguel.

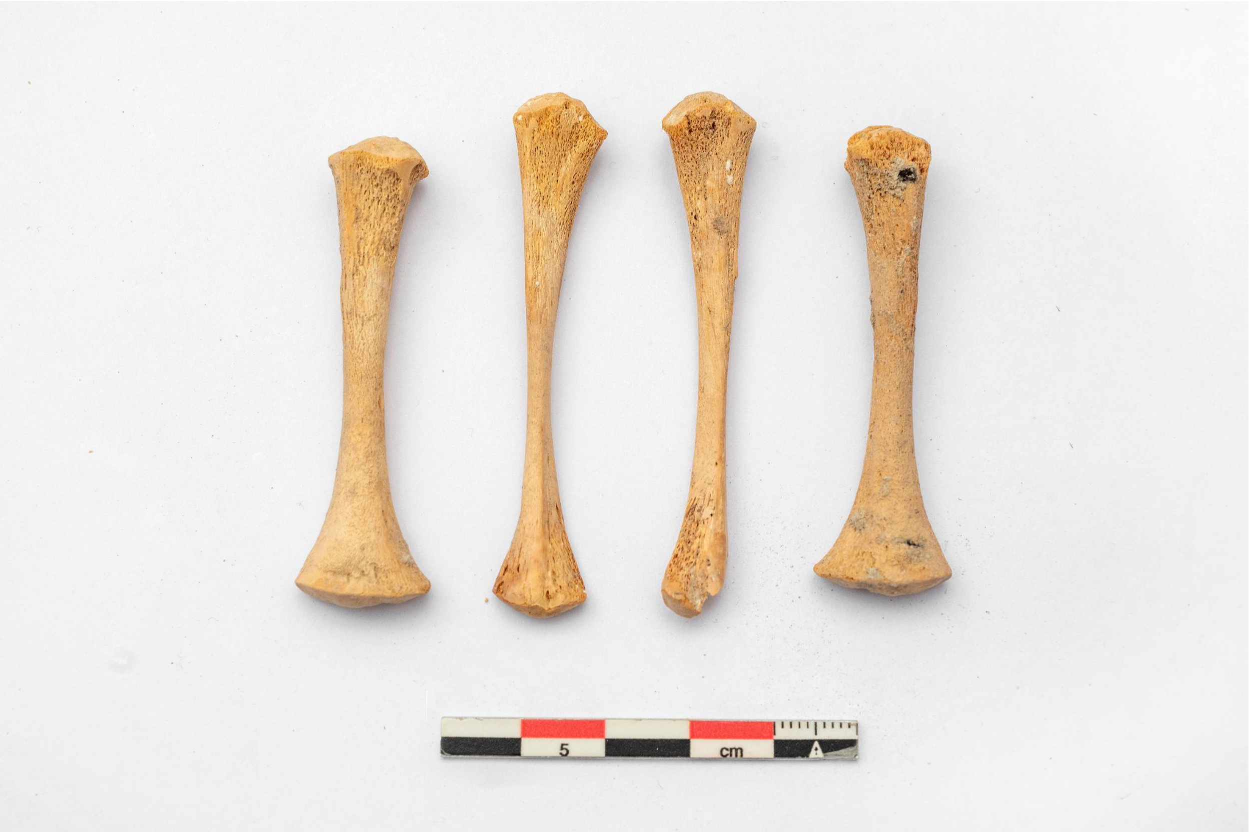

Supplementary Figure 11: anterior views of the humeri of CRU013 (Alto de la Cruz) indicating the decrease in the width of the diaphyses and metaphyses (middle) when compared to an individual of comparable age (left and right). The length of the two humeri indicates a gestational age of a term newborn. The width of the distal epiphyses and the width of the diaphysis for CRU013 indicate an approximate age of 28-30 weeks[18]. Photograph by Patxuka de Miguel.

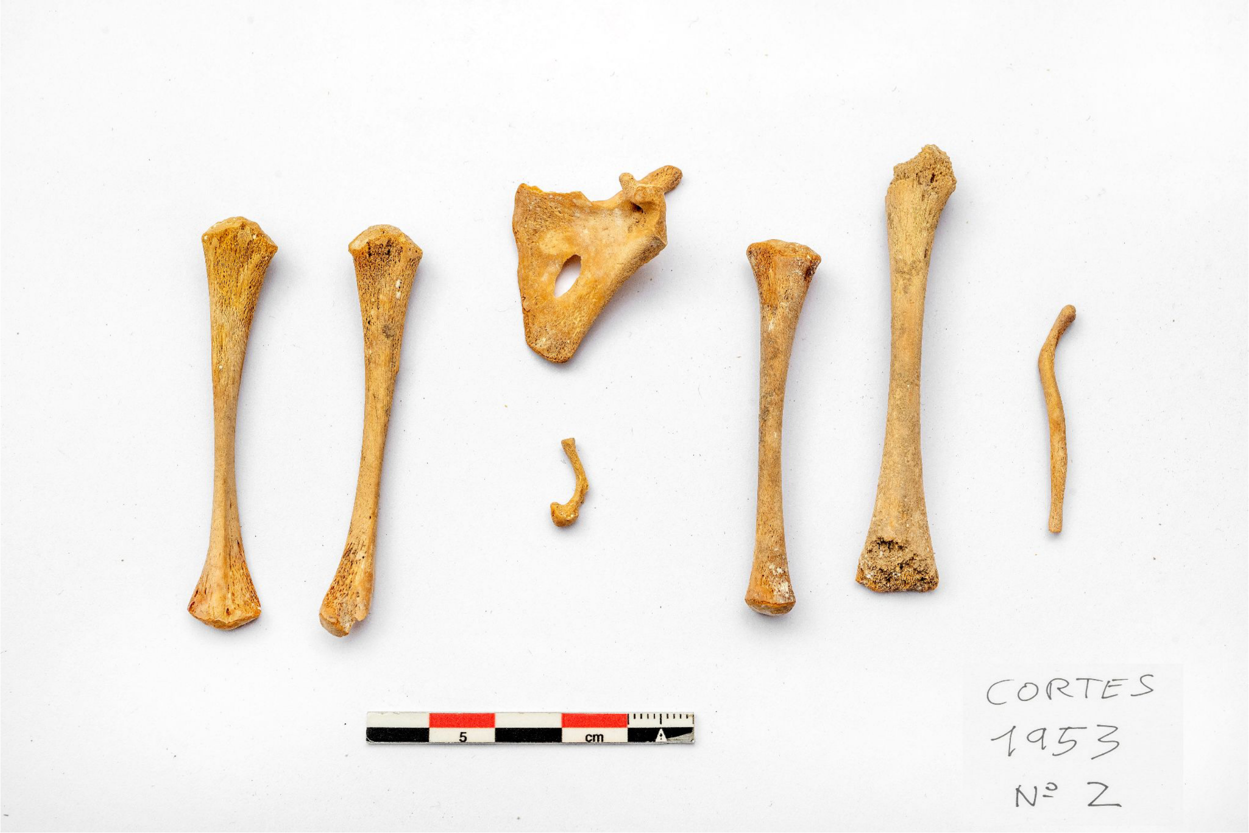

Supplementary Figure 12: The collection of bones which could be confidently attributed to CRU013 (Alto de la Cruz). These include (from left to right): the humeri (anterior view, above), left scapula (anterior view) and hemivertebra axis, left side (inferior view, below), left tibia (anterior view), right femur (anterior view), and incomplete and undetermined bone fragment. Photograph by Patxuka de Miguel.

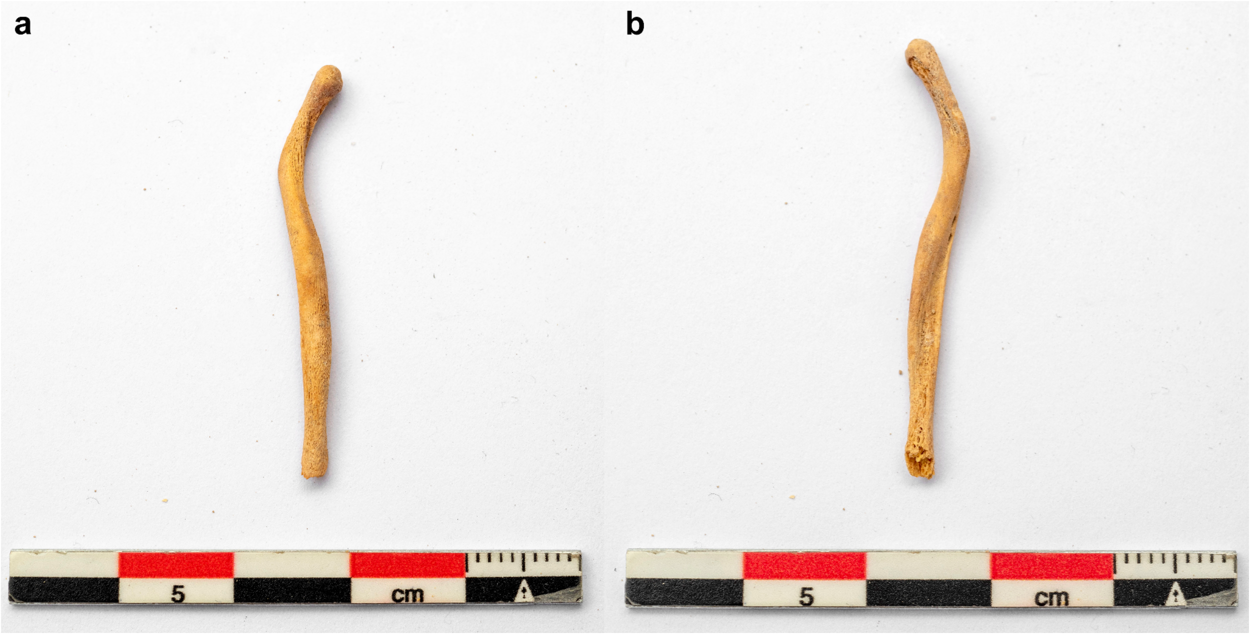

Supplementary Figure 13: incomplete and undetermined bone fragment from CRU013 (Alto de la Cruz). Photograph by Patxuka de Miguel.

References

1. Bengtsson H. *matrixStats: Functions that Apply to Rows and Columns of Matrices (and to Vectors)*. R package version 1.0.0 (2018).

2. Computing RFS. R: A language and environment for statistical computing. Vienna: R Core Team (2013).

3. Kircher M, Sawyer S, Meyer M. Double indexing overcomes inaccuracies in multiplex sequencing on the Illumina platform. *Nucleic Acids Res*. **40(1)**, e3 (2012).

4. Gansauge MT, Meyer M. Single-stranded DNA library preparation for the sequencing of ancient or damaged DNA. *Nat Protoc.* **8(4)**, 737-748 (2013).

5. Briggs AW, Stenzel U, Meyer M, Krause J, Kircher M, Pääbo S. Removal of deaminated cytosines and detection of in vivo methylation in ancient DNA. *Nucleic Acids Res*. **38(6)**, e87 (2009).

6. Renaud G, Stenzel U, Kelso J. leeHom: adaptor trimming and merging for Illumina sequencing reads. *Nucleic Acids Res.* **42(18)**, e141 (2014).

7. Li H, Durbin R. Fast and accurate short read alignment with BurrowsWheeler transform. *Bioinform*. **25(14)**, 1754-1760 (2009).

8. Телл Юнаците. Эпоха бронзы. Том II, Част первая. *Москва* **195**, 210-211 (2007).

9. Penske S, et al. Early contact between late farming and pastoralist societies in southeastern Europe. *Nature*. 1**9,** 1-8 (2023).

10. Mcsweeney K, Bacvarov K, Nikolov V, Andreeva D, C. Bonsall. *Infant burials in Early Bronze Age Bulgaria: a bioarchaeological appraisal of funerary behavior*. In: (Editors: Vassil Nikolov, Wolfram Schier). Der Schwarzmeerraum vom Neolithikum bis in die Früheisenzeit (6000600 v. Chr.), 391-401, Rahden/Westf (2016).

11. Eustratiou, K & Polychronakou-Sgouritsa, N. To Mykinaiko nekrotafeio stous LazaridesAiginas [Το Μυκηναϊκό νεκροταφείο στους Λαζάρηδες Αίγινας; The Mycenaean cemetery atLazarides, Aegina]. ArchDelt (Αρχαιολογικόν Δελτίον), 65-66, 2010-2011, 1-162.

12. Prevedorou E. A. bioarchaeological perspective on the human skeletal remains from the Mycenaean settlement and cemetery at Lazarides. To Mykinaiko nekrotafeio stous Lazarides Aiginas [Το Μυκηναϊκό νεκροταφείο στους Λαζάρηδες Αίγινας; The Mycenaen cemetery at Lazarides, Aegina] ArchDelt (Αρχαιολογικόν Δελτίον) 149–160 (2010–2011).

13. Maluquer de Motes J. El yacimiento hallstáttico de Cortes de Navarra: estudio crítico. 1. Diputacion Foral de Navarra, *Inst. "Principe de Viana"* (1954).

14. Maluquer de Motes J. El yacimiento hallstático de Cortes de Navarra. Estudio Crítico II.

Inst. “Principe de Viana” Pamplona (1958).

15. Maluquer de Motes J, Munilla Cabrillana G, Gracia Alonso F. Alto de la Cruz (Cortes, Navarra): campañas, 1986-1988. *Trabajos de Arqueología Navarra* **9**, 11-245 (1990).

16. Taracena Aguirre B. Excavaciones en Navarra. Cortes de Navarra. Los poblados de la edad del hierro, superpuestos en el“ Alto de la Cruz.” (1954).

17. Mercadal O, Campillo D, Pérez-Die M. Estudio paleoantropológico de los restos infantiles del Alto de la Cruz. Campañas 3/1987 y 4/1988. Maluquer de Motes, J., Gracia, F. y Munilla, G., Alto de la Cruz. Cortes de Navarra. Campañas. 1986;1988:219-43.

18. Armendáriz-Martija J, De-Miguel-Ibáñez MP. Los enterramientos infantiles del poblado de Las Eretas (Berbinzana): estudio paleoantropológico. *Trabajos de Arqueología Navarra* **19**, 5-44 (2006).

19. Hornborg E. Helsingin kaupungin historia, osa II. Ajanjakso 17211809. Helsingin kaupunki. 1950;

20. Lipkin S, Tranberg A, Kallio-Seppä T, Ruhl E. Preparing children’s burials in postmedieval Finland: Emotions awakened by sensory experiences. *Hist Archaeol*. **56(2)**,184-198 (2022).

21 Blackie D, Kuuliala J. Katsaus vammaisuuden historian tutkimukseen. *Hist Aikak.* **119(2)**, 156-164 (2021).

22. Stempfle N, Huten Y, Fredouille C, Brisse H, Nessmann C. Skeletal abnormalities in fetuses with Down's syndrome: a radiographic post-mortem study. *Pediatric radiology*. **29(9)**, 682-688 (1999).

23. Spitzer R, Rabinowitch JY, Wybar KC. A study of the abnormalities of the skull, teeth and lenses in mongolism. *CMAJ*. **84(11)**, 567 (1961).

24. Slomic AM, Bernier JP, Morissette J, Azouz EM. A craniometric study of Trisomy 21 (T 21). *Bulletins et mémoires de la Société d'anthropologie de Paris.* **2(2)**, 189-202 (1990).

25. Balkany TJ, Mischke RE, Downs MP, Jafek BW. Ossicular abnormalities in Down's syndrome. *Otolaryngol Head Neck Surg.* **87(3)**, 372-384 (1979).

26. Nordstrøm M, Retterstøl K, Hope S, Kolset SO. Nutritional challenges in children and adolescents with Down syndrome. *Lancet Child Adolesc Health*. **4(6)**, 455-464 (2020).

27. Cohen MM, Winer RA. Dental and facial characteristics in Down's syndrome (mongolism). *J Dent Res.* **44(1)**, 197-208 (1965).

28. Fazekas IG, Kósa F. *Forensic Fetal Osteology* (Akadémiai Kiadó, California, 1978).

29. Jeanty P. Fetal limb biometry. *Radiol*. **147(2)**, 601-602 (1983).
