# Supplementary material for: Cases of trisomy 21 and trisomy 18 among historic and prehistoric individuals discovered from ancient DNA
Source: Nat Commun. 2024 Feb 20;15:1294. doi: 10.1038/s41467-024-45438-1 (PMC10879165; doi:10.1038/s41467-024-45438-1)
Supplement: Supplementary file 3 — Reporting Summary [file 41467_2024_45438_MOESM3_ESM.pdf]

nature portfolio

Corresponding author(s): Dr Adam B Rohrlach
Dr Kay Prüfer

Last updated by author(s): 12-11-2023

Reporting Summary

Nature Portfolio wishes to improve the reproducibility of the work that we publish. This form provides structure for consistency and transparency in reporting. For further information on Nature Portfolio policies, see our Editorial Policies and the Editorial Policy Checklist.

Statistics

For all statistical analyses, confirm that the following items are present in the figure legend, table legend, main text, or Methods section.

n/a	Confirmed	
☐	☒	The exact sample size (*n*) for each experimental group/condition, given as a discrete number and unit of measurement
☐	☒	A statement on whether measurements were taken from distinct samples or whether the same sample was measured repeatedly
☐	☒	The statistical test(s) used AND whether they are one- or two-sided<br>*Only common tests should be described solely by name; describe more complex techniques in the Methods section.*
☒	☐	A description of all covariates tested
☒	☐	A description of any assumptions or corrections, such as tests of normality and adjustment for multiple comparisons
☐	☒	A full description of the statistical parameters including central tendency (e.g. means) or other basic estimates (e.g. regression coefficient) AND variation (e.g. standard deviation) or associated estimates of uncertainty (e.g. confidence intervals)
☐	☒	For null hypothesis testing, the test statistic (e.g. *F*, *t*, *r*) with confidence intervals, effect sizes, degrees of freedom and *P* value noted<br>*Give P values as exact values whenever suitable.*
☐	☒	For Bayesian analysis, information on the choice of priors and Markov chain Monte Carlo settings
☒	☐	For hierarchical and complex designs, identification of the appropriate level for tests and full reporting of outcomes
☒	☐	Estimates of effect sizes (e.g. Cohen's *d*, Pearson's *r*), indicating how they were calculated

Our web collection on statistics for biologists contains articles on many of the points above.

Software and code

Policy information about availability of computer code

Data collection	Data was processed using:<br>leeHom version 1.1.5<br>BWA version 0.7.12<br>samtools version 1.3
Data analysis	Data was processed using:<br>R version 4.2.0<br>matrixStats 1.0.0<br>Rfast 2.0.9<br>extraDistr 1.9.1<br>ggplot 2016

For manuscripts utilizing custom algorithms or software that are central to the research but not yet described in published literature, software must be made available to editors and reviewers. We strongly encourage code deposition in a community repository (e.g. GitHub). See the Nature Portfolio guidelines for submitting code & software for further information.

Data

Policy information about availability of data

All manuscripts must include a data availability statement. This statement should provide the following information, where applicable:

- Accession codes, unique identifiers, or web links for publicly available datasets
- A description of any restrictions on data availability
- For clinical datasets or third party data, please ensure that the statement adheres to our policy

Shotgun sequencing data for all seven detected trisomy cases have been deposited at the European Nucleotide Archive under the study accession number PRJEB71003. In addition, counts of reads mapping to each of the autosomes have been made available for all screened samples as a file with tab-separated-values (github.de/BenRohrlach/TrisomyAncientDNAStudy and as a supplementary file).

Research involving human participants, their data, or biological material

Policy information about studies with human participants or human data. See also policy information about sex, gender (identity/presentation), and sexual orientation and race, ethnicity and racism.

Reporting on sex and gender	We report only genetic sex, as defined by an individual having two copies of the X chromosome, or one copy of each of the X and Y chromosomes.
Reporting on race, ethnicity, or other socially relevant groupings	N/A
Population characteristics	N/A
Recruitment	N/A
Ethics oversight	N/A

Note that full information on the approval of the study protocol must also be provided in the manuscript.

Field-specific reporting

Please select the one below that is the best fit for your research. If you are not sure, read the appropriate sections before making your selection.

☐ Life sciences ☐ Behavioural & social sciences ☒ Ecological, evolutionary & environmental sciences

For a reference copy of the document with all sections, see nature.com/documents/nr-reporting-summary-flat.pdf

Ecological, evolutionary & environmental sciences study design

All studies must disclose on these points even when the disclosure is negative.

Study description	We screen 9,855 ancient human genomes for individuals which carry autosomal trisomies.
Research sample	We screened all samples from the in-house database at the Max Planck Institute for Evolutionary Anthropology for which shotgun data had been generated. Each sample is sequence data from a human individual.
Sampling strategy	We screened all samples from the in-house database for which general ethics clearance was granted. Since this is a screening study, no sample size calculations were made, and opportunistic sampling was employed.
Data collection	Data came from an in-house database of shotgun screening data. When an individual was flagged as positive for an autosomal trisomy, we contacted the archaeologists, osteologists and anthropologists from the original studies to contribute to descriptions of the skeletons and burial contexts. When this was not possible, we relied on the original publications and osteological reports.
Timing and spatial scale	Data was screened from as early as the European paleolithic (~45,000 years before present) to as recent as the early 1900s. Data comes from a global distribution.
Data exclusions	We excluded data for which ethics clearance was not granted.
Reproducibility	For individuals that were flagged as positive for an autosomal trisomy, we made sure that any additional libraries (additional shotgun data, or capture data) also exhibited the same behaviour and that ONLY the chromosome of interest was represented abnormally in the sample.
Randomization	N/A

Blinding	Blinding is not required as all "participants" have been dead for between ~100 years and ~45,000 years. Furthermore, these individuals are represented in the database by 6 character codes, and the researchers did not have prior knowledge of the 9,855 samples prior to screening.

Did the study involve field work? ☐ Yes ☒ No

Reporting for specific materials, systems and methods

We require information from authors about some types of materials, experimental systems and methods used in many studies. Here, indicate whether each material, system or method listed is relevant to your study. If you are not sure if a list item applies to your research, read the appropriate section before selecting a response.

Materials & experimental systems

n/a	Involved in the study	
☒	☐	Antibodies
☒	☐	Eukaryotic cell lines
☐	☒	Palaeontology and archaeology
☒	☐	Animals and other organisms
☒	☐	Clinical data
☒	☐	Dual use research of concern
☒	☐	Plants

Methods

n/a	Involved in the study	
☒	☐	ChIP-seq
☒	☐	Flow cytometry
☒	☐	MRI-based neuroimaging

Palaeontology and Archaeology

Specimen provenance	These data come from published studies or reports.
Specimen deposition	We do not report DNA sequence data for all 9,855 specimens, and instead for each specimen report the counts of reads mapping to each chromosome.
Dating methods	We do not report new radiocarbon dates, and the details for the radiocarbon dates for each sample can be found in the original cited studies.

☐ Tick this box to confirm that the raw and calibrated dates are available in the paper or in Supplementary Information.

Ethics oversight	No ethics oversight was required strictly, however we confirm that we followed established ethical guidelines for archaeogenetic research.

Note that full information on the approval of the study protocol must also be provided in the manuscript.
